# Reduced Retinoic Acid Receptor Beta (Rarβ) Affects Pancreatic β-Cell Physiology

**DOI:** 10.3390/biology11071072

**Published:** 2022-07-19

**Authors:** Anila Khalique, Abdul Khader Mohammed, Nujood Mohammed Al-khadran, Mutaz Al Gharaibeh, Eman Abu-Gharbieh, Waseem El-Huneidi, Nabil Sulaiman, Jalal Taneera

**Affiliations:** 1 Sharjah Institute for Medical Research, University of Sharjah, Sharjah 27272, United Arab Emirates; aabid@sharjah.ac.ae (A.K.); amohammed@sharjah.ac.ae (A.K.M.); u18100265@sharjah.ac.ae (M.A.G.); eabugharbieh@sharjah.ac.ae (E.A.-G.); welhuneidi@sharjah.ac.ae (W.E.-H.); 2 Department of Basic Medical Sciences, College of Medicine, University of Sharjah, Sharjah 27272, United Arab Emirates; u18105590@sharjah.ac.ae; 3 Department of Clinical Sciences, College of Medicine, University of Sharjah, Sharjah 27272, United Arab Emirates; 4 Department of Family Medicine, College of Medicine, University of Sharjah, Sharjah 27272, United Arab Emirates; nsulaiman@sharjah.ac.ae

**Keywords:** *RARα*, *RARγ*, *RARβ*, diabetes, insulin secretion, glucose uptake, insulin, human islets, INS1 cells, vitamin A

## Abstract

**Simple Summary:**

Vitamin A is one of the important micronutrients involved in various biological functions. Numerous studies have suggested a link between vitamin A (retinoic acid) and type 2 diabetes (T2D). However, the functional role of vitamin A receptors (RARα, β, and γ) in β-pancreatic cells is not clear yet. In this study, we performed bioinformatics and functional experiments in human islets and INS-1 cells in order to evaluate the potential role of RARβ on insulin secretion and pancreatic β-cell function.

**Abstract:**

Various studies have suggested a link between vitamin A (VA), all-*trans*-retinol, and type 2 diabetes (T2D). However, the functional role/expression of vitamin A receptors (*Rarα*, *β,* and *γ*) in pancreatic β-cells is not clear yet. Accordingly, we performed a series of bioinformatics, molecular and functional experiments in human islet and INS-1 cells to evaluate the role of *Rarβ* on insulin secretion and pancreatic β-cell function. Microarray and RNA-sequencing (RAN-seq) expression analysis showed that *RARα*, *β,* and *γ* are expressed in human pancreatic islets. RNA-seq expression of *RARβ* in diabetic/hyperglycemic human islets (HbA1c ≥ 6.3%) revealed a significant reduction (*p* = 0.004) compared to nondiabetic/normoglycemic cells (HbA1c < 6%). The expression of *RARβ* with *INS* and *PDX1* showed inverse association, while positive correlations were observed with *INSR* and HbA1c levels. Exploration of the T2D knowledge portal (T2DKP) revealed that several genetic variants in RARβ are associated with BMI. The most associated variant is rs6804842 (*p* = 1.2 × 10^−25^). Silencing of *Rarβ* in INS-1 cells impaired insulin secretion without affecting cell viability or apoptosis. Interestingly, reactive oxygen species (ROS) production levels were elevated and glucose uptake was reduced in *Rarβ*-silenced cells. mRNA expression of *Ins1*, *Pdx1*, *NeuroD1*, *Mafa*, *Snap25*, *Vamp2*, and *Gck* were significantly (*p* < 0.05) downregulated in *Rarβ*-silenced cells. For protein levels, Pro/Insulin, PDX1, GLUT2, GCK, pAKT/AKT, and INSR expression were downregulated considerably (*p* < 0.05). The expression of NEUROD and VAMP2 were not affected. In conclusion, our results indicate that *Rarβ* is an important molecule for β-cell function. Hence, our data further support the potential role of VA receptors in the development of T2D.

## 1. Introduction

Type 2 diabetes mellitus (T2D) is a multifactorial and heterogeneous metabolic disorder characterized by a higher glucose level due to insufficient insulin production, action, or both. The disease’s prevalence is increasing globally and is expected to affect more than 550 million people in the next 10 years (www.idf.org, accessed on 16 May 2022). As our understanding of the pathogenesis of T2D has been dramatically increased, the interaction between genetic susceptibility and lifestyle, including diet, are well-established as the main factors in the occurrence of the disease [1].

Dietary components including carbohydrates, fats, and vitamins are well-recognized to increase the prevalence of T2D [2,3]. VA is a lipophilic dietary metabolite found in plants as carotenoids or in animals as retinyl esters [4]. Typically, VA is stored in the liver and secreted into the blood circulation, where it binds to retinol-binding protein (RBP4) [5]. In addition, it is well established that VA is a crucial biomolecule that regulates cellular differentiation and immunity and is essential for reducing oxidative stress [6]. 

In humans, VA plays an essential role in pancreatic endocrine development during the early stages, where deficiency can decrease β cell mass and impair glucose tolerance in adulthood [7,8,9,10,11,12]. Moreover, a decrease in pancreatic VA level in the adult mouse pancreas disturbed the pancreatic β cell mass ratio to α cells, leading to the altered serum level of insulin and glucagon [13,14]. Currently, there is some epidemiological evidence supporting the association between VA and the risk of T2D in humans [15,16,17]. Furthermore, studies on animal models have shown that VA deficiency in rats impaired biphasic insulin release. However, a large dose of retinoic acid restored insulin secretion in pancreatic islets [18]. Additionally, it was demonstrated that VA elevated the production of, insulin, glucokinase (GCK) activity, and GLUT2 expression; however, on the other side, it also inhibited the proliferation of β- pancreatic cells [19].

At the transcriptional level, VA is mediated via the binding and activation of several retinoic-acid receptors (RAR-α, β, and γ) along with three retinoid-X receptors (RXR-α, β, and γ) [15,20]. Transgenic mice overexpressing a dominant-negative Rarα mutant (RARdn) controlling Pdx1 promoter exhibited glucose-stimulated insulin secretion impairment and downregulated mRNA expression levels of Glut2 and Gck as decreases in β-cell mass [21]. Rarβ knockout in embryonic stem cells has been shown to diminish the pancreatic endocrine differentiation process and reduce the expression of Pdx1, Gcg, Iapp, and Ins1 compared to control cells [22]. Another study showed that overexpression of Rarβ in RINm5F cells increased insulin secretion in the presence of all-trans-retinoic acid (ATRA) [23].

Together, these data indicate that RAR signaling is essential to maintaining β-cell function and mass. However, the role of Rarβ and γ in insulin secretion is not clear. Additionally, the expression of RARs in murine pancreatic cells are well documented; however, expression in human pancreatic islets is not mature [22,24]. Thus, in the current study, we utilized RNA-seq data to evaluate the expression profile of RARs in human pancreatic islets +/− diabetes and their correlations with HbA1c as well as other key β-cell function genes. In addition, we performed several in vitro functional studies on INS-1 cells to describe the role of Rarβ in insulin secretion and β-pancreatic cell function.

## 2. Materials and Methods

### 2.1. mRNA Microarray and RNA-Sequencing Data

Human pancreatic islet RNA-seq expression data were retrieved from a publicly available database (GEO, accession number: GSE50398). RNA-seq data set includes pancreatic islets from 77 cadaver donors, which includes 50 nondiabetic (HbA1c < 6%) and 25 diabetic (HbA1c ≥ 6%) donors [25]. For microarray expression data, we utilized our previous microarray data that are publicly available (GEO, accession number: GSE41762). The microarrays (GeneChip^®^ HG Waltham, MA, USA) were implemented according to the Affymetrix standard protocol [26]. Pancreatic islets were isolated from 67 non-diabetic/normoglycemic donors (age range 59 ± 10, BMI 25.9 ± 3.5, HbA1c level 5.5 ± 0.5, 37 males and 30 females).

### 2.2. Analysis of Genetic Variants in Rarβ for the Association with T2D

The type 2 diabetes (T2D) knowledge data portal (https://t2d.hugeamp.org (accessed on 16 May 2022)), which consists of 310 datasets and 339 traits, was used to analyze the association of genetic variants within RABβ and genetic information that is linked with T2D and associated traits.

### 2.3. Cell Culture

The rat pancreatic β-cells (INS-1, passage #51) were cultured in RPMI 1640 as described previously [27]. The cells were cultured at 37 °C in a humidified incubator in a fixed atmosphere of 5% CO_2_, and a fresh growth medium was used to replenish cells every 2–3 days until 80–90% confluence was obtained.

### 2.4. RNA Interference

Pancreatic β-cells (INS-1) were silenced as in a previously described method [27,28] using Lipofectamine™ 3000 transfection substance and siRNA sequences against Rarb (s128322 and s128323) (Thermo Fisher Scientific, Waltham, MA, USA). A negative control silencer (Thermo Fisher Scientific, Waltham, MA, USA) was used. INS-1 cells were cultured in RPMI 1640 at 37 °C in a humidified incubator in a fixed atmosphere of 5% CO_2_ for 72 h. The concentration of siRNA is 40 nM in 24-well cell culture plates (200,000 cells/well). Transfected cells were washed twice with 1 mL secretion assay buffer (SAB) at pH 7.2 having 2.8 mM glucose and then incubated for 2 h in a 2 mL new SAB (with 2.8 mM glucose). Next, an additional incubation step for 1 h was performed in 1 mL SAB with 2.8/16.7 mM glucose.

### 2.5. Insulin Secretion Measurement

After 48 h of transfection, GSIS (glucose-stimulated insulin secretion) was investigated. Transfected INS1 cells were incubated in SAB buffer containing 0.2% BSA at pH 7.2 with 2.8 mM glucose for 2 h. GSIS was performed by incubating cells in 1 mL SAB containing 2.8 mM glucose along with the secretagogue mentioned previously [27,28]. Insulin secretion and content were measured using a rat-insulin ELISA kit (Mercodia, Uppsala, Sweden). To determine the insulin content, total protein in the cells was extracted and diluted at 1:250 and normalized to the total protein amount in the cells.

### 2.6. Western Blot Analysis

Western blot analysis was performed as in a previously described protocol [27,28] with the following primary and secondary antibodies; VAMP2-Cat. #13508 (anti-rabbit), Insulin Receptor-β-Cat. #23413 (anti-rabbit), and INSULIN-Cat. #8138 (anti-mouse) were purchased from CST (Cell signaling Technology, Danvers, MA, USA). NEUROD1-Cat. #ab213725 (anti-rabbit), PDX1-Cat. #ab47267 (anti-rabbit), and GCK-Cat. #ab37796 (anti-rabbit) were purchased from Abcam (Cambridge, UK). GLUT2-Cat. #A12307 (anti-rabbit) was purchased from ABclonal (Waltham, MA, USA). The β-actin-Cat. #A5441 (anti-mouse) endogenous control was purchased from Sigma, St. Louis, MO, USA. The secondary HRP-linked anti-mouse-Cat. #7076 and anti-rabbit-Cat. #7074 were purchased from Cell Signaling Technology. Protein bands were quantified and analyzed by using the Bio-Rad Image Lab software.

### 2.7. Methylthiazolyldiphenyl-Tetrazolium Bromide (MTT) Cell Viability Assay

Briefly, 2 × 10^5^ INS-1 cells were seeded in a 96-well plate for 24 h and transfected as previously described in the Section 2. At 48 hours, 10 μL of MTT (Sigma, St. Louis, MO, USA) was added and incubated for 3–4 h at 37 °C and absorbance was read at 570 nm [29] using a microplate reader (Crocodile Control Software, Winston Salem, NC, USA).

### 2.8. Quantitative-PCR

Total RNA was extracted by RNA Kit (Invitrogen, Waltham, MA, USA) and quantity/quality was measured on Nanodrop (Thermo Fisher, Waltham, MA, USA). RevertAid H Minus First Strand cDNA Synthesis Kit was used to produce cDNA (Thermo Fisher, Waltham, MA, USA). Gene expression quantification was performed by TaqMan assay (Thermo Fisher, Waltham, MA, USA) in qPCR Quantstudio3 machine from Applied Biosystems (Waltham, MA, USA) with specific primer probes for Ins2 (Rn01774648_g1), Ins1 (Rn02121433_g1), Pdx1 (Rn00755591_ m1), Mafa (Rn00845206_g1) Insr (Rn00690703_m1), Glut2 (Rn00563565_m1), Gck (Rn00561265_m1), and Hprt1 (Rn01527840_m1). Expression of Snap25, Vamp2, NeuroD, Rarβ, Rarγ, and Rarα were perfumed by using the following SYBR-Green primers: Snap25 (forward: GGCGTTTGCTGAATGACAAC; reverse: CAGAGCCTGACACCCTAAGA), Vamp2 (forward: TGGTGGACATCATGAGGGTG; reverse: GCTTGGCTGCACTTGTTTCA), NeuroD (forward: CCCTAACTGATTGCACCAGC; reverse: TGCAGGGT AGTGCATGGTAA), RARβ (forward: CCA GAG CAA GAC ACA CCA TGA CT; reverse; CAT CCA TTT CCA AAG GCA GGA), RARα (forward: ACT TGT CCC TTT GCC CCT CT; reverse: CTT TCG TAC ATC TTG CCC CG), RARγ (forward: TGC AGC TGA AGA TCA CCC C; reverse: GTA ACC AGA TCC AGG CCC C). Hprt (forward primer: TTGTGTCATCAGCGA AAGTGG; reverse primer: CACAGGACTAGAACGTCTGCT) was used to normalize the gene expression. The relative gene expression was measured by 2^−ΔΔCt^ method.

### 2.9. Apoptosis Assay

Transfected INS-1 cells were cultured in a complete RPMI for 24 hours as previously described [27,28]. Next, cells were washed with PBS and suspended in 0.5 mL solution of Annexin-V Buffer (BD Biosciences, Franklin, NJ, USA). In the next step, 5 μL of Propidium Iodide (PI) and Annexin-V were used to stain the transfected cells for 10 min in the dark, followed by flow cytometry (BD, FACS, Becton Dickinson, Franklin, NJ, USA).

### 2.10. Glucose Uptake Assay

Glucose analog 2-NBDG, from Invitrogen (Cat. #N13195), was used to assess glucose uptake according to the manufacturer’s procedure. Briefly, siRarβ cells were incubated with 100 µM/mL of 2-NBDG (dissolved in ethanol) and incubated for 1 h in a humidified incubator at 37 °C. Next, the harvested cells were washed two times with ice-cold PBS, then centrifuged at 1800 rpm for 5 min at 4 °C. Finally, cells were re-suspended in 250 µL of ice-cold PBS. Stained cells were immediately quantified by flow cytometry (FACS Aria^TM^ III) and analyzed by FlowJo_v10.8.1 software (BD, Williamson Way Ashland, OR, USA).

### 2.11. ROS Production

To measure the H_2_O_2_, a commercially available assay kit (ROS-Glo H_2_O_2_ Assay-Promega) was used. The manufacturer’s guidelines were followed when performing the H_2_O_2_ assay. Briefly, 15,000 INS1 cells were seeded in a 96-well plate overnight; after 24 h transfection, 20 µL of H_2_O_2_ substrate was added to every well and incubated at 37 °C in a humidified incubator in a fixed atmosphere of 5% CO_2_ for 4 h. After incubation, 0.1 mL of ROS-Glo detection reagent was added to the experimental wells and was kept for 20 min at room temperature. Immediately after, the fluorescent intensity was measured by using a luminometer plate reader.

### 2.12. Statistical Analysis

Student *t*-tests or nonparametric Mann–Whitney tests were used for differential expression analysis between diabetic versus non-diabetic islets. The correlation analyses were performed by nonparametric Spearman’s correlation test. Statistical analyses were performed on GraphPad Prism, V8 Software, San Diego, CA, USA). In all analyses, differences were significant at * *p* < 0.05, ** *p* < 0.01 or *** *p* < 0.001.

## 3. Results

### 3.1. Expression of RARs in Human Pancreatic Islets

**Figure 1:**
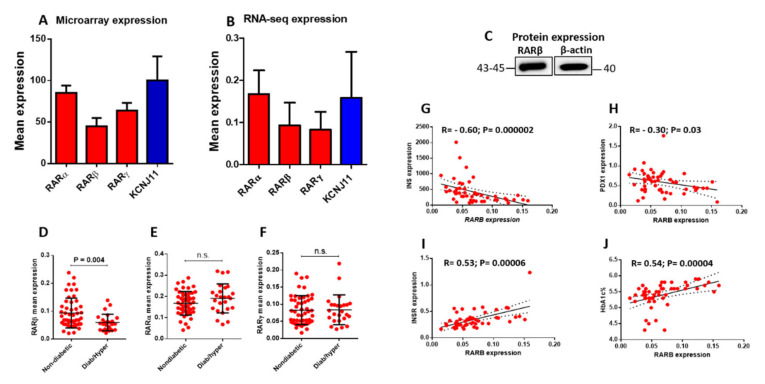
Expression profile of RARs in human pancreatic islets. (**A**) microarray mean gene expression of RARα, β, γ, and KCNJ11 in nondiabetic/normoglycemic human islets (n. of donors = 45) as determined on Human Gene 1.0 ST array. (**B**) RNA-seq gene expression of RARα, β, γ, and KCNJ11 in nondiabetic/normoglycemic human islets (n. of donors = 50). (**C**) Western blot expression of RARβ was obtained from non-diabetic human islets (*n* = 1; purchased from Prodo Lab. Inc, Maple Plain, MI, USA). Differential expression patterns of RARβ (**D**), RARα (**E**), and RARγ (**F**) in nondiabetic/normoglycemic (n. of donors = 50) versus diabetic/hyperglycemic (n. of donors = 25) human islets. Spearman’s expression correlation of RARβ with INS (**G**), PDX1 (**H**), INSR (**I**), and HbA1c levels (**J**) (n. of donors = 50). Expression levels are presented as mean ± SD. Full western blot figures are available in Supplementary File.

Microarray and RNA-seq expression data from human pancreatic islets were used to describe the expression profile of RARs. As shown in Figure 1A,B, RARs were presented in human pancreatic islets in both analyses, with RARα having the highest expression level compared to RARβ and RARγ. To evaluate the expression levels of RARs in human islets, we used the β-cell functional gene KCNJ11 [30] as a relative reference value. RARα revealed a comparable expression level to KCNJ11, while RARβ and RARγ were lower (Figure 1A,B). In addition, RARβ expression was further confirmed at the protein level by using nondiabetic human pancreatic islets (*n* = 1) (Figure 1C).

Next, we investigated whether RARs are differentially expressed in diabetic/hyperglycemic islets. As illustrated in Figure 1D–F, RARβ exhibited a significant expression reduction (*p* < 0.05) in diabetic islets (HbA1c ≥ 6.0%) compared to nondiabetic islets (HbA1c < 6%) (Figure 1D). Expression levels of RARα and RARγ were not affected (Figure 1E,F). Additionally, co-expression correlation with RARβ showed a negative association with INS and PDX1 (Figure 1G,H), whereas positive correlations were observed with INSR and HbA1c levels (Figure 1I,J). No correlations of RARβ expression with glucose transporter 1/2 (GLUT1/GLUT2), RARα, RARγ, body mass index (BMI), or gender (data not shown) were observed.

Further, we evaluated whether RARβ contains any genetic variants associated with T2D or related traits in the T2D knowledge portal. Interestingly, we found a strong association between several genetic variants in RARβ and BMI in the GIANT UK Biobank GWAS. Accordingly, 103 genetic variants were shown to pass the significance threshold at *p*  <  1 × 10^−8^ for the association with BMI, with rs6804842 being the strongest associated genetic variant. A list of the top 10 associated genetic variants in RARβ with BMI are shown in Table 1.

### 3.2. Silencing of Rarβ Impairs Insulin Secretion, ROS Production and Glucose Uptake

**Figure 2:**
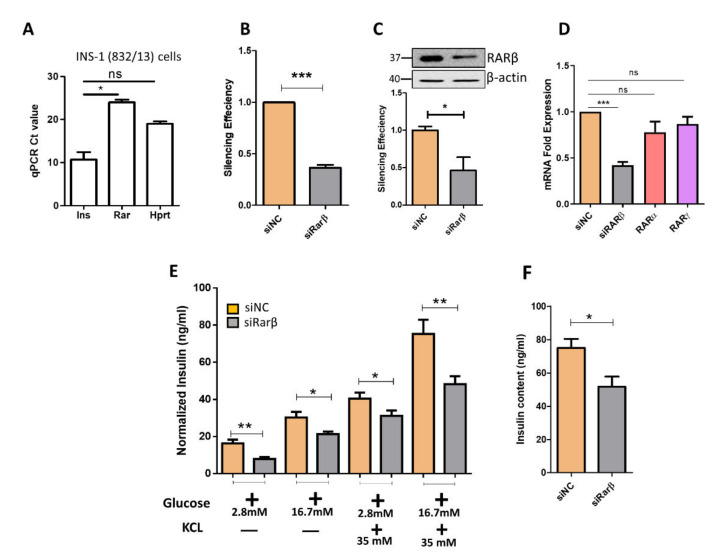
Impact of *Rarβ* silencing on insulin secretion. (**A**) qPCR expression analysis of *Rarβ* and Insulin genes (*Ins*) in INS-1 cells. (**B**) Silencing efficiency of *Rarβ* mRNA expression 48 h after transfection as determined by q-PCR. (**C**) Silencing efficiency of *Rarβ* protein expression 48 h after transfection as determined by Western blot expression analysis in INS-1 cells relative to the endogenous control β-actin (upper panel). Fold changes in the intensity of the Western blot band are shown in the lower panel. (**D**) qPCR expression analysis of RARα and RARγ in siRarβ cells or negative control cells 48 h after transfection. (**E**) Glucose-stimulated insulin secretion (normalized to protein) in response to low glucose (2.8 mM) and high glucose (16.7 mM), along with 35 mM potassium chloride (KCl), in si*Rarβ* cells compared to negative control cells. (**F**) Insulin content measurements normalized to total protein content in *siRarβ* compared to the negative control. Data were acquired from three independent experiments. In all analyses, differences were significant at—*: *p* < 0.05, **: *p* < 0.01 and ***: *p* < 0.001. Bars represent mean ± SD. Full western blot figures are available in Supplementary File.

**Figure 3:**
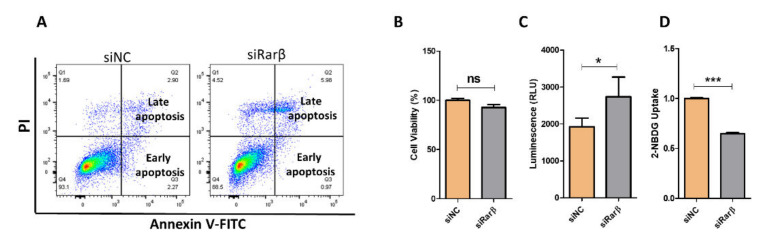
Impact of siRarβ on apoptosis, ROS production, and glucose uptake. (**A**) Apoptosis levels in Rarβ-silenced cells compared to negative control cells are determined by flow cytometry analysis. (**B**) Cell viability percentage evaluated by MTT assay in Rarβ-silenced cells. (**C**) ROS production in Rarβ-silenced cells compared to control cells. (**D**) Glucose uptake efficacy in Rarβ-silenced cells compared to control cells. Results are obtained from 3 independent experiments. *: *p* < 0.05 and ***: *p* < 0.001. Bars represent mean ± SD.

To explore the effect of Rarβ ablation on insulin secretion and β-cell function, we first investigated the expression level of Rarβ in beta pancreatic cells. As shown in Figure 2A, the expression Ct value of Rarβ in INS-1 was 25 compared to 14 for the Insulin (INS) gene or 18 for Hprt. Next, we silenced Rarβ expression in INS-1 cells using two different siRNAs sequences (pool). Assessment of silencing efficiency 48 h post-transfection by qPCR and Western blotting showed ~65% decrease in Rarβ expression (*p* < 0.05) relative to the negative control (Figure 2B,C). In addition, analyzing the impact of Rarβ silencing on the mRNA expression of Rarα and Rarγ showed no significant effect (Figure 2D). Rarβ-silenced cells revealed a significant decrease in GSIS at basal level (2.8 mM glucose; (~50%, *p* < 0.05) and at stimulation level (16.7 mM glucose; ~35%, *p* < 0.05) after 1 h stimulation compared with control cells (Figure 2E). Stimulating Rarβ-silenced cells with 35 mM KCl (depolarizing agent) for 1 h showed a significant decrease in insulin secretion (*p* < 0.05) (Figure 2E). Insulin content measurements showed a 20% reduction in Rarβ-silenced cells compared to control cells (*p* < 0.05) (Figure 2F). Then, we examined whether the observed reduction of insulin secretion in Rarβ-silenced cells was attributed to disrupting cell viability and apoptosis. As shown in Figure 3A, the apoptosis levels tested by annexin-V staining revealed no significant effect (*p* > 0.05) on the proportion of apoptotic cells (~7%; early and late apoptosis) out of the total number of Rarβ-silenced cells compared to 5% with the control cells. These results were further confirmed by the cell viability test, which showed no significant (*p* > 0.05) effect of siRarβ on cell viability compared to the negative control cells (Figure 3B). To this end, we examined the effect of siRarβ on ROS production and glucose uptake. Figure 3C shows ROS measurements representing a marked elevation (~22%; *p* < 0.05), as evidenced by the increased positive rate of fluorescence and mean fluorescence intensity in siRarβ cells compared to control cells. On the other side, a substantial reduction in glucose uptake (~30%; *p* < 0.05) was observed in siRarβ cells compared to control cells (Figure 3D).

### 3.3. Silencing of Rarβ in INS-1 Cells Influences Key β-Cells

**Figure 4:**
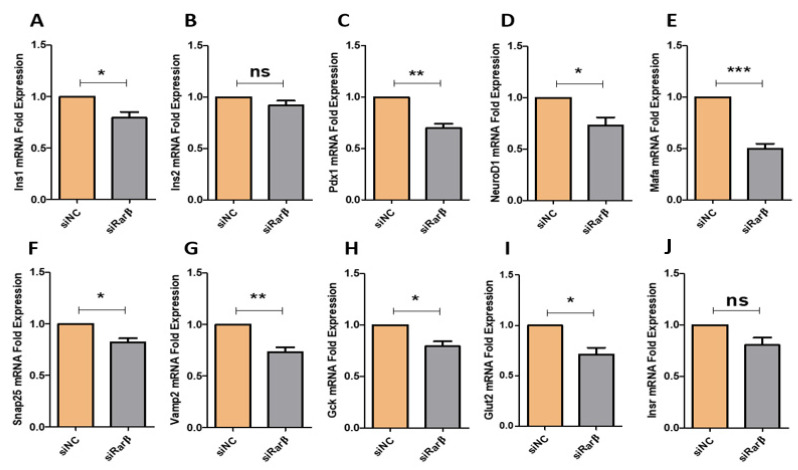
Impact of *Rarβ*-silencing on β-cell function genes at mRNA level. qPCR was performed in si*Rarβ* or negative control cells at 48 h after transfection. (**A**) *Ins1*, (**B**) *Ins2,* (**C**) *Pdx1,* (**D**) *NeuroD1,* (**E**) *Mafa,* (**F**) *Snap25*, (**G**) *Vamp2*, (**H**) *Gck*, (**I**) *Glut2,* and (**J**) *Insr*. Data acquired from 3 independent experiments. *: *p* < 0.05, **: *p* < 0.01 and ***: *p* < 0.001. Bars represent mean ± SD.

**Figure 5:**
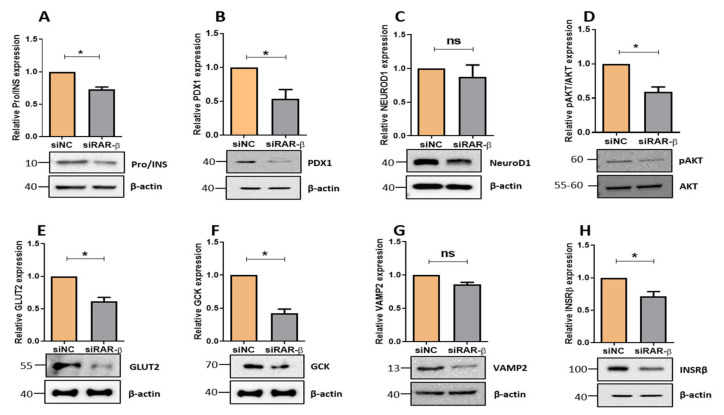
Impact of *Rarβ*-silencing on β-cell function genes at protein level. Western blot analysis of Pro/insulin (**A**), PDX1 (**B**), NEUROD1 (**C**), p-AKT/AKT (**D**), GLUT2 (**E**), GCK (**F**), VAMP2 (**G**), and INSβ (**H**) normalized to the endogenous control protein β-actin. Corresponding fold changes in the intensity of the Western blot are shown below each blot. Data are obtained from three independent experiments. *p*-value. *: *p* < 0.05. Error bars show mean ± SD. Full western blot figures are available in Supplementary File.

To elucidate the impact of *Rarβ* silencing on genes involved in pancreatic β-cell function, we analyzed the mRNA expression of insulin biosynthesis genes. As illustrated in Figure 4, the expression of *Ins1* was significantly (*p* < 0.05) reduced (Figure 4A), whereas *Ins2* was not affected (Figure 4B). Expression levels of the β-cell transcription factors showed a significant decrease in *Pdx1*, *NeuroD1,* and *Mafa* (musculoaponeurotic fibrosarcoma oncogene family, protein A) (*p* < 0.05) (Figure 4C,E). Gene expression analysis of Snap25 (Synaptosome-associated protein 25) and Vamp2 (Vesicle-associated membrane protein 2) showed downregulation (*p* < 0.05) in Rarβ-silenced cells compared to negative control cells (Figure 4F,G). Moreover, the expression levels of genes involved in glucose sensing and insulin signaling (*Gck*, *Glut2)* were significantly reduced in siRar*β* cells compared to the negative control (*p* < 0.05) (Figure 4H,I). However, the insulin receptor (*Insr*) was also downregulated non-significantly (Figure 4N). Protein expression analysis showed a significant downregulation of Pro/Insulin (~25%; *p* > 0.05) (Figure 5A), PDX1(~50%; *p* > 0.05) (Figure 5B), and p-AKT/AKT (~40%; *p* > 0.05) (Figure 5D). Moreover, expression of GLUT2 (~40%; *p* < 0.05) (Figure 5E), GCK (~60%; *p* < 0.05) (Figure 5F), and INSRβ (~30%; *p* < 0.05) (Figure 5H) was reduced in Rarβ-silenced cells compared to control cells. The expression levels of NEUROD1 and VAMP2 were not affected by the silencing of *Rarβ* in INS-1 cell (Figure 5C,G).

## 4. Discussion

Several studies have demonstrated that retinoids and VA possess anti-obesity and anti-lipogenic activities via transcriptional regulation of multiple relevant genes, mainly in the liver and adipose tissue [12,31,32] However, less work has been done on pancreatic β cells. The current study showed that RARα, β, and γ are expressed in human pancreatic islets. As the focus of this work was on RARβ, we demonstrated that expression of RARβ is downregulated in diabetic/hyperglycemic islets and correlated with HbA1c, HbA1c, INS, and INSR genes. Furthermore, the silencing of Rarβ in INS-1 cells showed impaired insulin secretion (without any cytotoxic effect), influencing the β-cell function genes, increasing ROS production, and reducing glucose uptake. Together, these presented data suggest that Rarβ plays an essential role in β-cell function.

Several points are worth mentioning; first, the expression of RARs is well-documented in animal models or cell lines [10,21,23], but less work has been conducted on human pancreatic islets. Thus, this present study is the first that systematically established the expression of RARs in human pancreatic islets using two different powerful approaches (microarray and RAN-seq). Herein, we focused on the expression of RARβ. The expression of RARβ was validated at the mRNA and protein levels in human islets (Figure 1). In contrast to previous reports that showed no/very low expression of Rarβ in β-cells [33,34], we demonstrated by qPCR and Western blotting that Rarβ is expressed in the INS-1 cell line (Figure 2).

Secondly, the reduced expression levels of RARβ in diabetic islets is a novel finding (Figure 1D). Whether such reduction contributes to impaired insulin release is not clear. However, in that case, the finding may increase the possibility that agents targeting RARβ may characterize a novel therapeutic to enhance the β-pancreatic cell function and boost insulin secretion. Importantly, our data do not rule out whether such observed reduction in RARβ expression is causative of defective insulin release or a consequence of long-term glucotoxicity. A previous study reported that short-term exposure of human islets to higher glucose concentrations did not affect the expression of RARβ [35].

Thirdly, the co-expression correlation of RARβ with the Insulin gene or HbA1c levels was surprising. Considering the expression of RARβ is downregulated in diabetic islets, we expected the expression RARβ would positively correlate with Insulin gene expression and negatively with HbA1c, but that was not the case. Possible explanations for this finding are a change in mRNA expression of the tested genes due to long-term hyperglycemia status or epigenetic regulations that control RARβ gene expression [36]. Additionally, the long-term diabetes-lowering medications taken by the donors might have a role in regulating HbA1c levels and enhancing insulin release.

As determined, silencing of the Rarβ gene in INS-1 cells impaired insulin release without affecting cytotoxicity or apoptosis (Figure 2E). Expression of Rarβ has been reported during pancreas development [37] and plays a major role in endothelial stem cell (ESC) differentiation to pancreatic endocrine cells. Although it is well known that retinoic acid signaling can regulate cell proliferation, differentiation, and apoptosis [18], the silencing of Rarβ in INS-1 cells showed no substantial differences in the cell viability’s apoptosis rate, which rules out their involvement in the impairment of insulin secretion. The inability of Rarβ-silencing to induce apoptosis could be attributed to a compensation mechanism by other RAR genes to reward the lack of Rarβ. Baker and his colleagues demonstrated that activation of retinoic acid receptors regulates glucose metabolism by inhibiting intracellular ROS production and improving insulin resistance [38]. Our results also demonstrated that ROS generation significantly increases in Rarβ-silenced cells compared to the negative control cells, indicating that retinoic receptors resist ROS generation.

To gain more insight into the molecular mechanisms of how Rarβ diminished insulin secretion, we investigated the expression of several β-pancreatic cell genes mainly involved in insulin biosynthesis, glucose sensing, insulin action, and insulin release. Previously, the retinoic acid-response element was anticipated to stimulate insulin mRNA levels in human islets [39]. In line with these data, the observed downregulation of mRNA and protein expression analysis of insulin (Figure 4 and Figure 5) indicated that Rarβ signaling was involved in regulating insulin gene expression.

Furthermore, expression assessment at the mRNA or protein levels of the β-cell transcription factors showed significantly decreased Pdx1, Mafa, and Neurod1 (Figure 4). However, only Pdx1 was persistently reduced at the protein level. Pdx1 is an important transcription factor, especially in the early determination of pancreatic progenitors and the maturation/dysfunction of β-cell [40,41]. In line with our data, it has been reported that the absence of Rarβ leads to a delay in Pdx1 expression [22]. ChIP analyses proposed a RARE (retinoic acid response element) located upstream of the transcription start site of Pdx1 in teratocarcinoma cells (F9) [22]. Thus, we can speculate that ablation of Rarβ reduced the Pdx1 expression pattern, which resulted in β-cell dysfunction and reduced insulin secretion. As noticed, the transcriptional change of NeuroD1 was not mirrored by protein expression. This could be explained by the post-transcriptional regulations that might affect mRNA stability and translation rate.

Glut2 and Gck are two major players in glucose-sensing machinery that respond to physiological blood glucose changes in β-pancreatic cells. Several studies reported that any defect in glucose-sensing machinery would impair insulin secretion, which causes severe hyperglycemia [42,43]. In the current study, mRNA expression levels of Glut2 and Gck were found significantly downregulated in Rarβ-silenced cells (Figure 4). Similarly, protein level analysis revealed a dramatic reduction of GLUT2 and GCK (Figure 5). In line with these data, it has been reported that the expression of Glut2 and Gck were downregulated in islets from RARdn mice [21].

Interestingly, the reduced expression of GLUT2 was accompanied by decreased glucose uptake efficacy, which is an essential driving factor for insulin release (Figure 3). Thus, our data suggest that ablation of Rarβ expression affects the β-cell capacity for glucose sensing, which probably contributes to the decrease in insulin response. However, insulin secretion in response to KCl was impaired in Rarβ-silenced cells. Therefore, it seems that Rarβ impact occurs downstream of the depolarization of the β-cell membrane, as reported previously in Rarα [21]. The decreased expression of Insr in Rarβ-silenced cells is an exciting finding as this could lead to a defect in insulin secretion. It has been demonstrated that ablation of Insr in β-pancreatic cells can impair insulin secretion [42]. In addition, several studies have established Snap25 and Vamp2 as important factors in the fusion process of insulin granules and plasma membranes [44,45]. Our data showed downregulated mRNA expression of Snap25 and Vamp2 but not at protein levels. Whether Rarβ suppressed insulin secretion through insulin granule trafficking and fusion pathways requires further investigation. Together, the data indicate that Rarβ has a promising role in insulin sensing by decreasing the expression of glucose-sensing and insulin-signaling regulators.

In mammals, there are three isoforms of Akt (1, 2, 3) which play divergent functions. However, it is well known that Akt2 is a crucial element in insulin signaling [46]. Akt activation requires complete phosphorylation of Thr308, Ser473, and Ser474. Bastien and his colleague demonstrated that the presence of retinoic acid (VA) phosphorylation of Ser22 decreased the proliferation of murine F9 cells [47]. Our data showed that silencing of Rarβ downregulated pAKT/AKT levels in INS-1 cells.

Although the strength of this study is the combination of genetics data through bioinformatics analysis with functional data, it is worth noting that this study has certain limitations. We used only an in vitro model, which cannot mimic human physiology. Second, the RNA-sequencing data addressed the expression of RARs in human pancreatic islets instead of sorted β-cells or other endocrine cells. Third, we focused only on Rarβ; other RARs might similarly affect pancreatic β-cell function. It will be of great interest to perform further investigations to dissect the role of Rarβ in other tissues, such as muscles and liver, and the association with glucose metabolism.

## 5. Conclusions

In conclusion, the current data suggest that VA (all-trans-retinol) and its receptors could be important for glucose metabolism and pancreatic β-cell dysfunction. In addition, our data also raise the possibility of using Rarβ as a drug target for T2D.

## Figures and Tables

**Table 1:** Association of most significant variant (BMI) in RARβ obtained from the T2D knowledge portal.

ID	Reference Allele	Alternate Allele	*p*-Value	Beta
rs6804842	A	G	1.2 × 10^−25^	▲0.0156
rs10510554	T	C	5.6 × 10^−23^	▲0.0156
rs7619139	T	A	2.6 × 10^−21^	▲0.0154
rs4858697	A	G	1.6 × 10^−20^	▲0.0149
rs1609783	G	A	2.7 × 10^−20^	▲0.0149
rs12632128	G	T	8.3 × 10^−20^	▲0.0146
rs6767671	G	T	2.4 × 10^−19^	▲0.0147
rs4858696	C	T	2 × 10^−18^	▲0.0146
rs9847186	G	A	3.3 × 10^−18^	▼−0.0140
rs4353774	A	C	3.3 × 10^−18^	▲0.0143

## Data Availability

Not applicable.

## Supplementary Materials

The following supporting information can be downloaded at: https://www.mdpi.com/article/10.3390/biology11071072/s1. Full western blot figures.
