# Supplementary material for: Reduced Retinoic Acid Receptor Beta (Rarβ) Affects Pancreatic β-Cell Physiology
Source: Biology (Basel). 2022 Jul 19;11(7):1072. doi: 10.3390/biology11071072 (PMC9312298; doi:10.3390/biology11071072)

Replicate 1

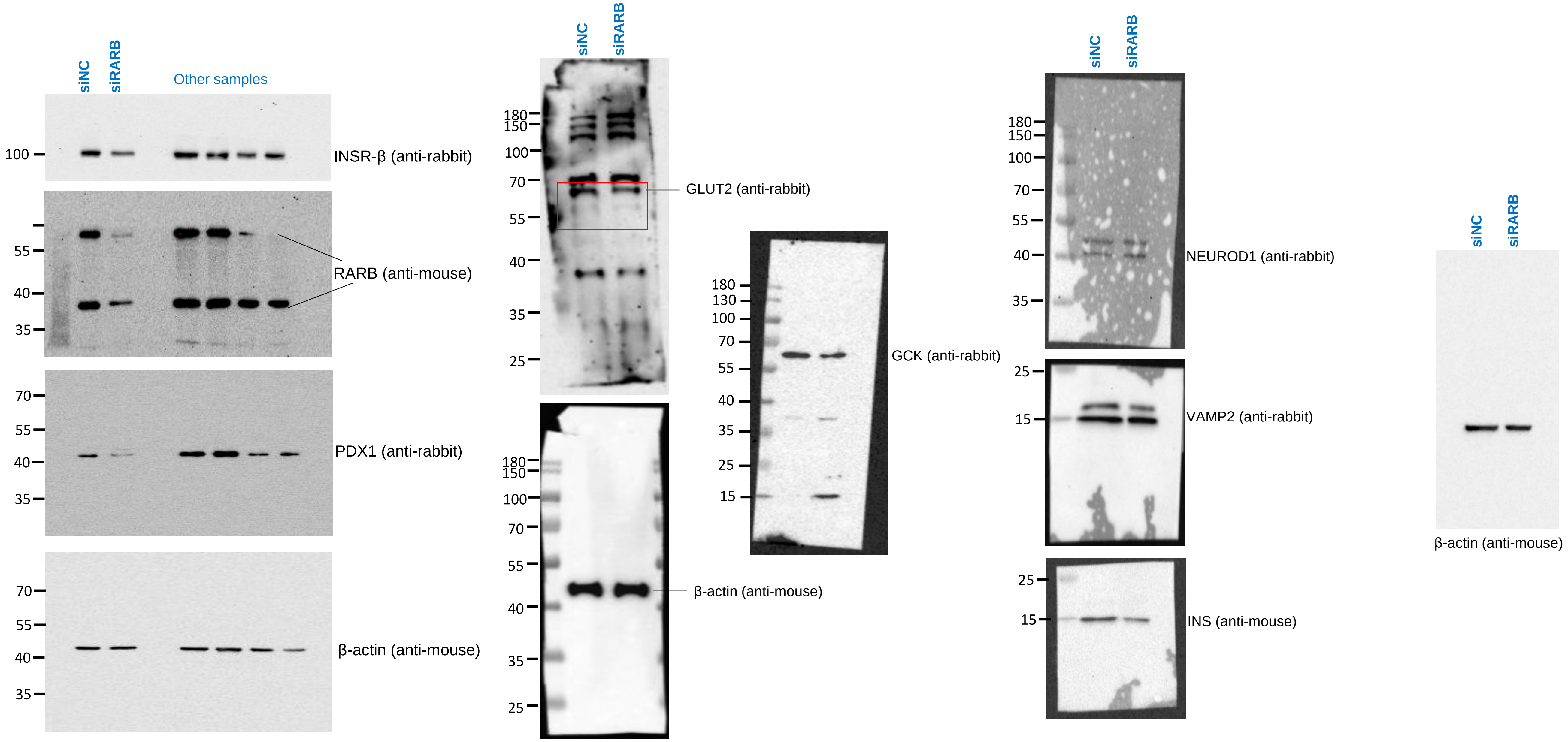
siNC
siRARB
Other samples
INSR-β (anti-rabbit)
RARB (anti-mouse)
PDX1 (anti-rabbit)
β-actin (anti-mouse)
GLUT2 (anti-rabbit)
β-actin (anti-mouse)
GCK (anti-rabbit)
NEUROD1 (anti-rabbit)
VAMP2 (anti-rabbit)
INS (anti-mouse)
β-actin (anti-mouse)
180
150
130
100
70
55
40
35
25
15

Replicate 2

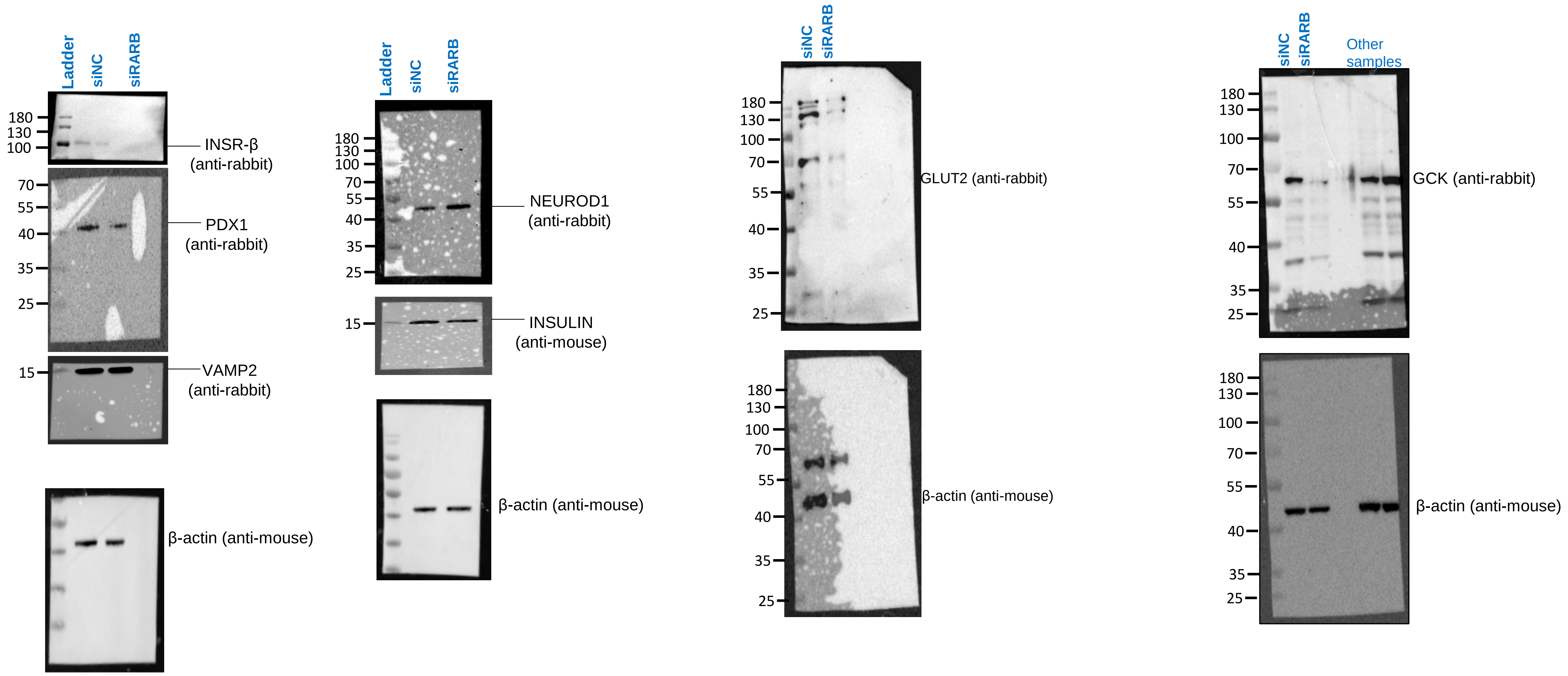

Ladder
siNC
siRARB
180
130
100
INSR-β
(anti-rabbit)
70
55
40
35
25
PDX1
(anti-rabbit)
15
VAMP2
(anti-rabbit)
β-actin (anti-mouse)
Ladder
siNC
siRARB
180
130
100
70
55
40
35
25
NEUROD1
(anti-rabbit)
15
INSULIN
(anti-mouse)
β-actin (anti-mouse)
siNC
siRARB
180
130
100
70
55
40
35
25
GLUT2 (anti-rabbit)
180
130
100
70
55
40
35
25
β-actin (anti-mouse)
siNC
siRARB
Other samples
180
130
100
70
55
40
35
25
GCK (anti-rabbit)
180
130
100
70
55
40
35
25
β-actin (anti-mouse)

Replicate 3

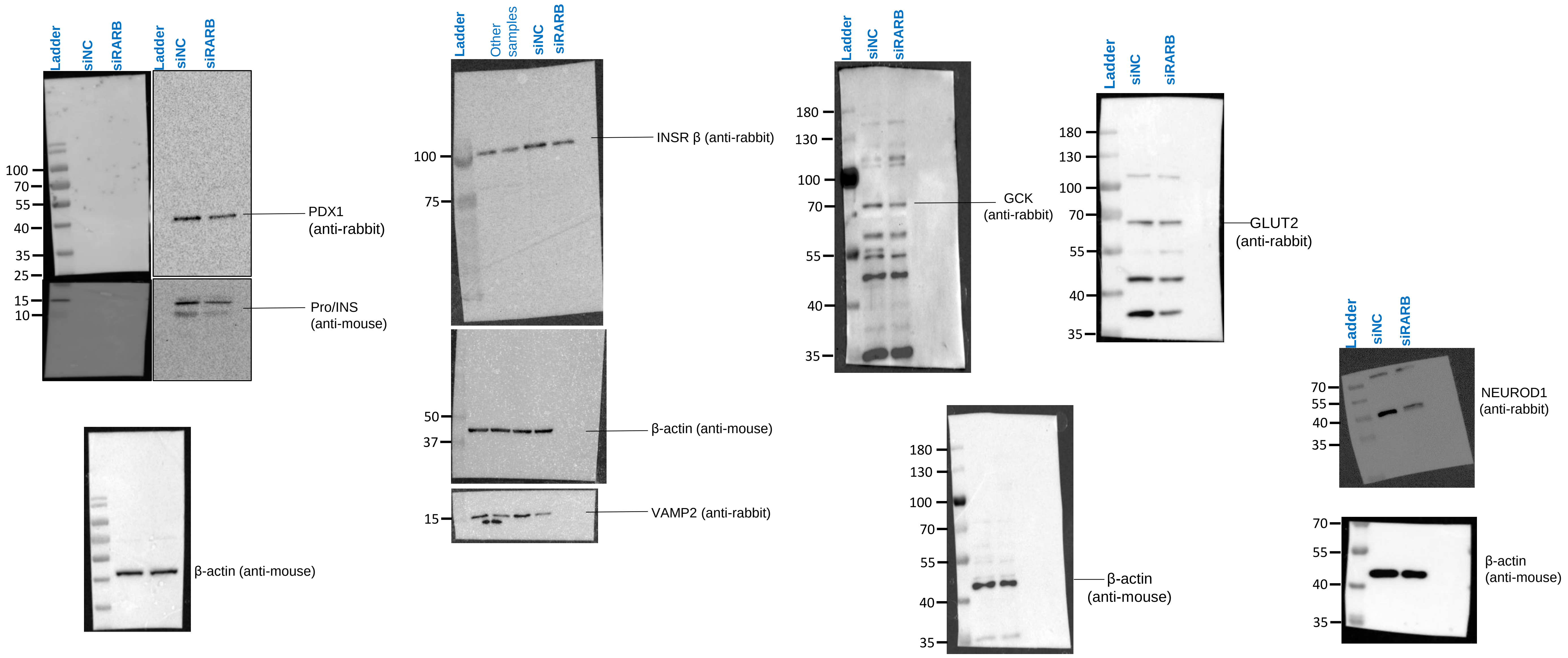
Ladder
siNC
siRARB
Ladder
siNC
siRARB
100
70
55
40
35
25
15
10
PDX1
(anti-rabbit)
Pro/INS
(anti-mouse)
β-actin (anti-mouse)
Ladder
Other samples
siNC
siRARB
100
75
INSR β (anti-rabbit)
50
37
β-actin (anti-mouse)
15
VAMP2 (anti-rabbit)
Ladder
siNC
siRARB
180
130
100
70
55
40
35
GCK
(anti-rabbit)
Ladder
siNC
siRARB
180
130
100
70
55
40
35
GLUT2
(anti-rabbit)
180
130
100
70
55
40
35
β-actin
(anti-mouse)
Ladder
siNC
siRARB
70
55
40
35
NEUROD1
(anti-rabbit)
70
55
40
35
β-actin
(anti-mouse)

pAKT and total AKT protein Expression

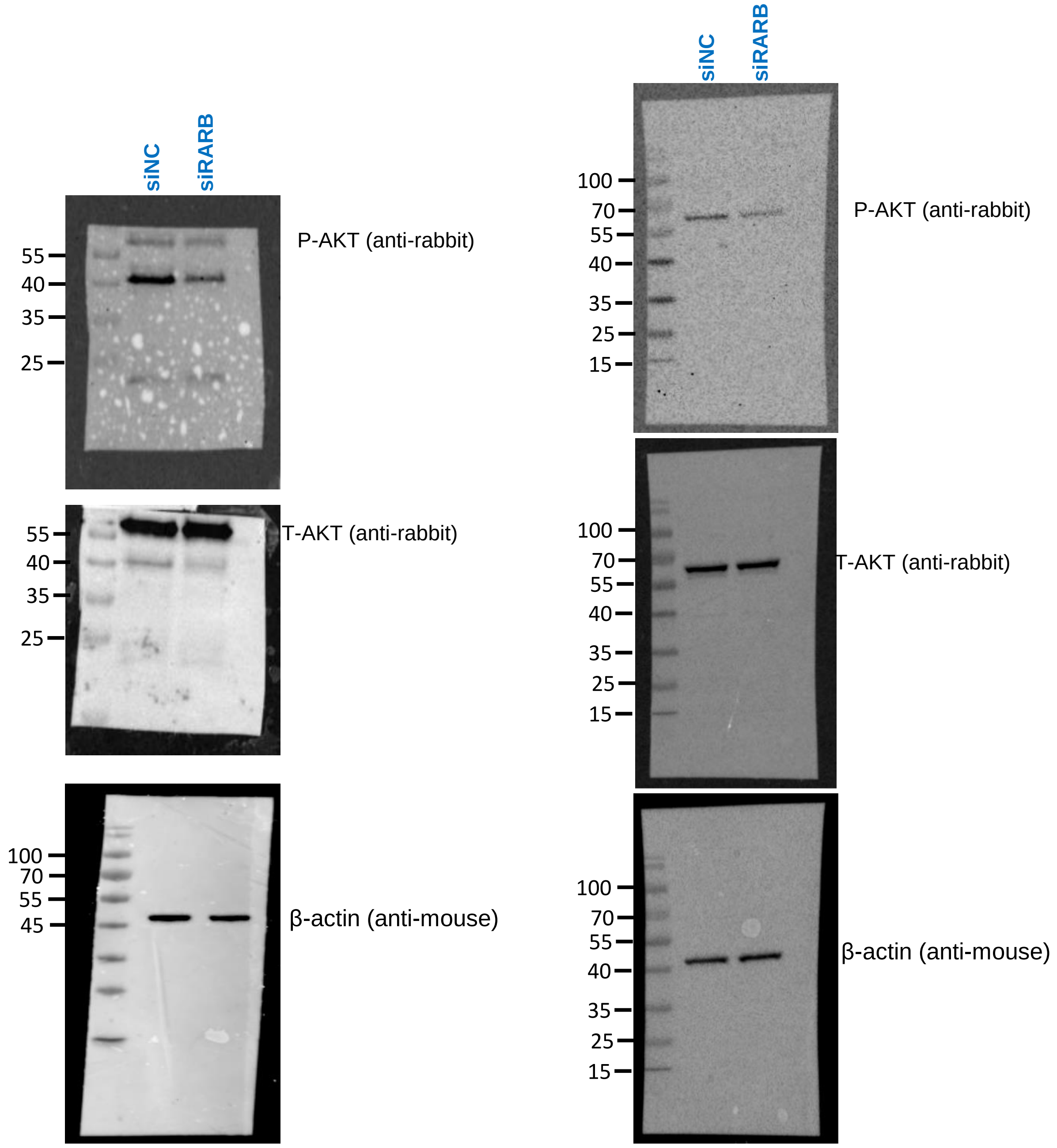

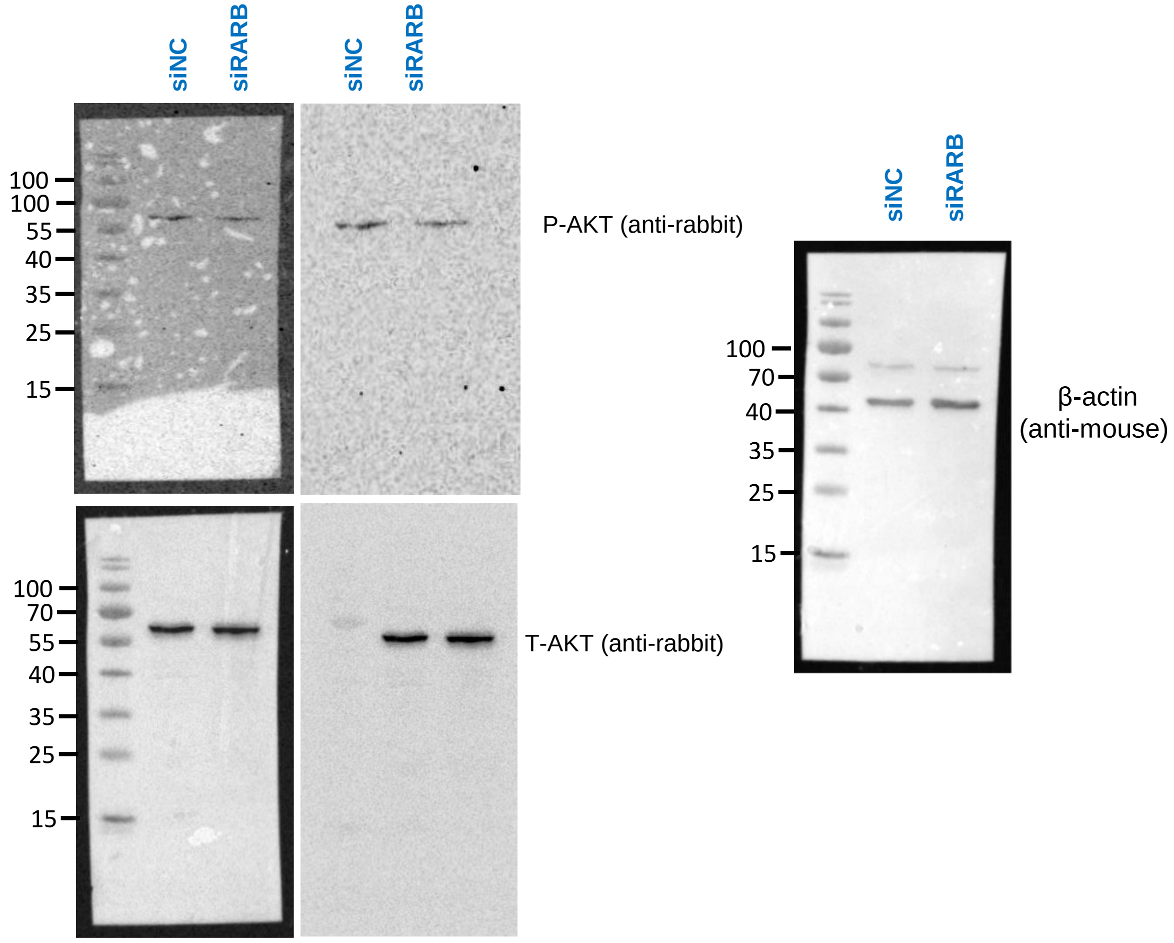

RARB Protein Expression

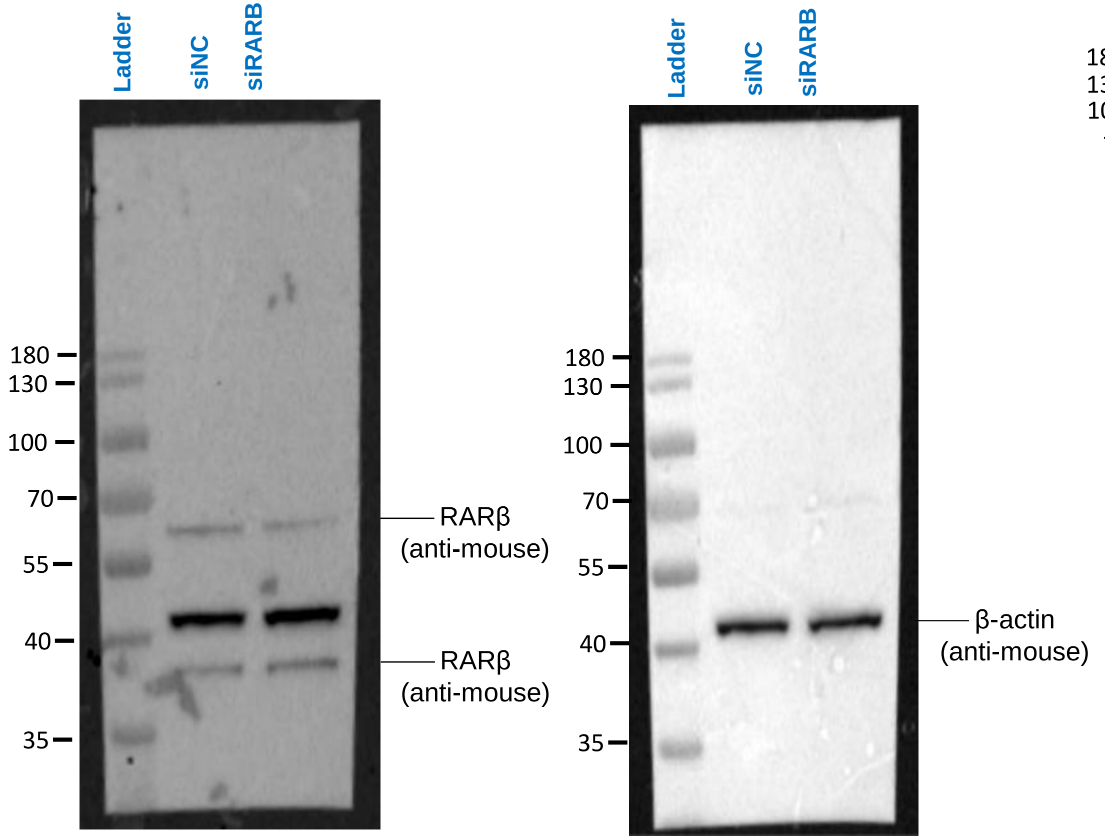

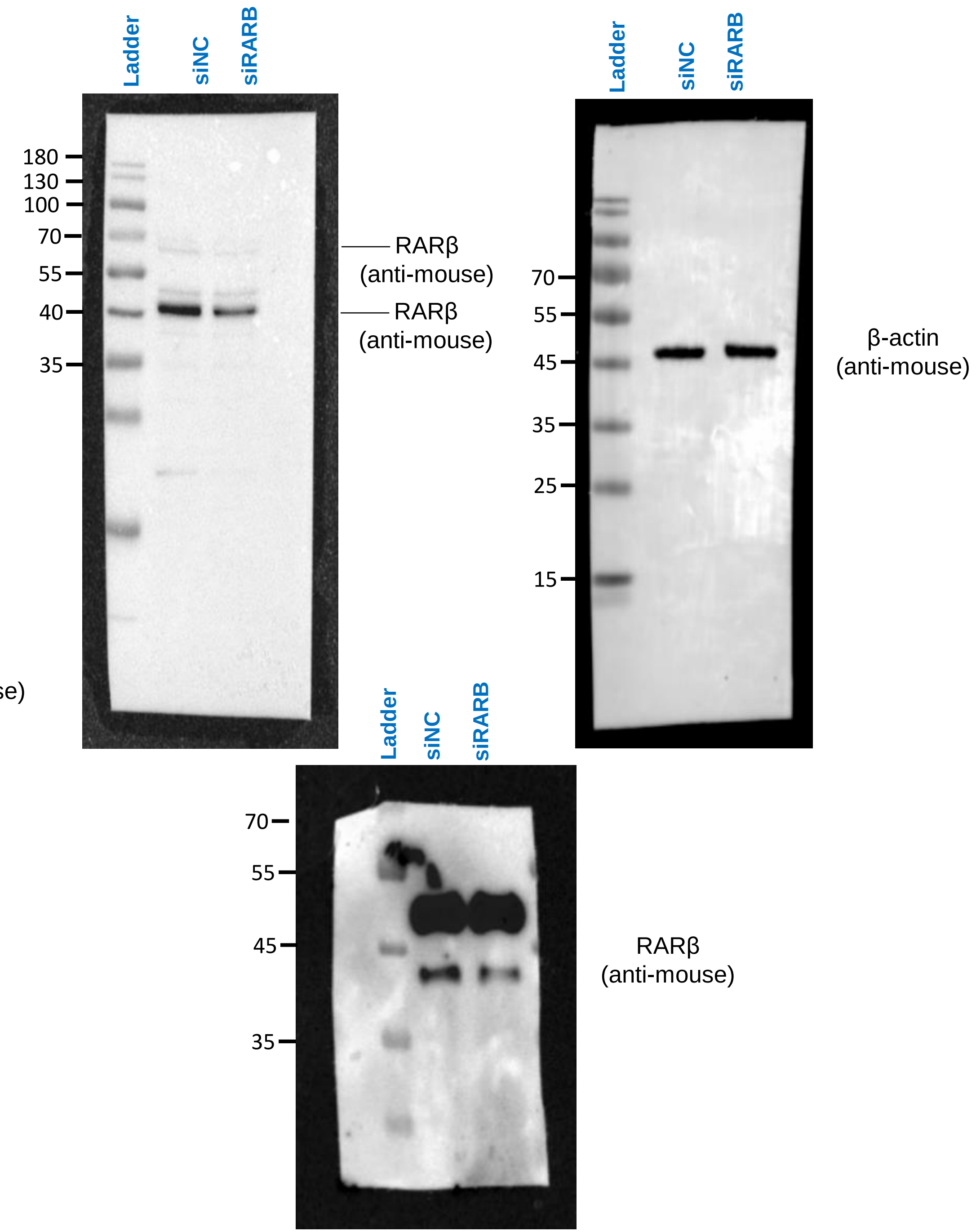

RARB Expression in human islets

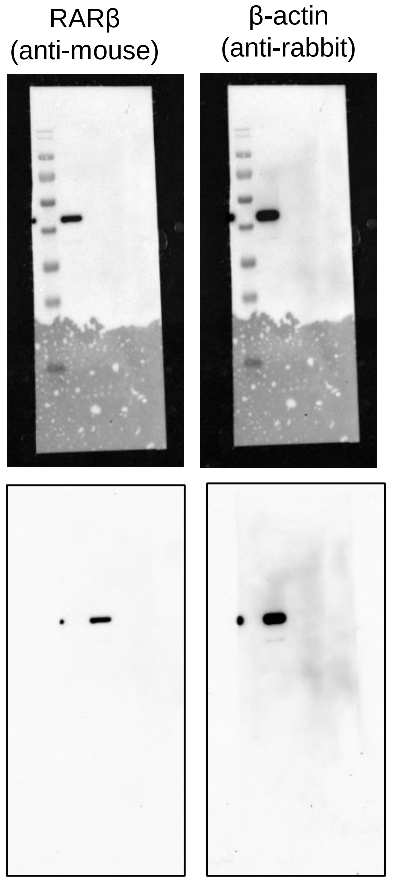

Supplement: Supplementary file 1 [file biology-11-01072-s001.zip › biology-1784228-supplementary.pdf]
